# Academic exam periods and ultra-processed food consumption: evidence from supermarket transactions in a Colombian University

**DOI:** 10.3389/fpsyg.2025.1726856

**Published:** 2026-01-27

**Authors:** Daniel Parra, Neha Khandpur, Laura Guerrero Sánchez, Juan Carlos Londoño Roldan, Jeremy C. Young

**Affiliations:** 1 Department of Business Administration, School of Economics and Management, Pontificia Universidad Javeriana, Bogotá, Colombia; 2 Division of Human Nutrition and Health, Wageningen University, Wageningen, Netherlands; 3 Inter-American Development Bank, Washington, DC, United States; 4 Business School, Tecnologico de Monterrey, Ciudad de Mexico, Mexico

**Keywords:** academic stress, difference-in-differences, eating behavior, NOVA classification, nutrition policy, ultra-processed foods

## Abstract

**Introduction:**

Academic stress can change eating behavior and often leads to higher consumption of unhealthy foods. This study examines whether exam periods affect students' purchases of ultra-processed foods, using objective purchase data rather than self-reports.

**Methods:**

We analyze point-of-sale transaction data from a university supermarket in Colombia. We use a difference-in-differences design. Students are the treatment group and university staff are the control group. We compare ultra-processed food purchases across pre-exam, exam, and post-exam periods.

**Results:**

During exam weeks, students increase their purchases of ultra-processed foods by 12.9 percent relative to non-exam periods, with statistical significance at *p* < 0.05. No significant changes appear among non-students. The effects vary across time, with changes observed before exams, during exams, and after exams.

**Discussion:**

The results provide causal evidence that exam-related stress or time constraints increase demand for ready-to-heat, ultra-processed foods. By relying on transaction data, this study overcomes limitations of self-reported measures. The findings highlight clear temporal patterns in stress-related or convenience-driven food choices and suggest relevant implications for campus nutrition policies and stress management interventions in academic settings.

## 1 Introduction

Stressful periods often affect what people choose to eat, changing both the quantity and quality of food consumed. While some individuals experience a reduced appetite, others develop intense cravings for calorie-dense and unhealthy foods (Gibson, 2012). This behavioral response has implications for health and well-being, particularly among younger individuals who frequently face predictable stressors such as academic examinations. A crucial concern is that ultra-processed foods (hereafter, UPFs) have become dominant in the global food system and are a major contributor to the worldwide rise in obesity. These products are characterized as durable, convenient, and highly palatable food and beverage formulations containing few or no whole ingredients (Monteiro et al., 2018). More importantly, UPF consumption has been associated with several chronic diseases, including increased incidence of dyslipidemia, overweight and obesity, hypertension, and cancer (Canella et al., 2014; Mendonça et al., 2017; Fiolet et al., 2018; Chen et al., 2023).

The term ultra-processed foods comes from the Nova classification, which categorizes foods and beverages based on the type and extent of industrial processing they experience. This system defines four groups: Group 1 includes unprocessed or minimally processed foods such as edible parts of plants and animals, fungi, algae, and water, while Group 4 consists of ultra-processed foods, products primarily made from food substances and additives, often subjected to extensive industrial processing (e.g., hydrogenation, extrusion) to produce convenient and marketable items (Monteiro et al., 2018). The processes and ingredients used to manufacture ultra-processed foods are designed to create highly profitable (using low-cost ingredients and emphasizing branding), convenient (ready-to-consume), and hyper-palatable products with long shelf lives, which tend to displace other Nova food groups, particularly unprocessed or minimally processed foods (Monteiro et al., 2018).

Previous research has documented a positive correlation between unhealthy food consumption and negative emotional states (for a meta-analysis, see Hill et al., 2022). Gibson (2012) report that between one-third and two-thirds of surveyed individuals eat more when stressed, and that one-quarter to one-half engage in comfort eating, a behavior more prevalent among women and individuals with obesity. Errisuriz et al. (2016) show that psychological distress affects dietary intake among Korean adolescents, while Smaira et al. (2021) provide evidence that Brazilian women increased their consumption of UPF products during the COVID-19 pandemic. Similarly, Lopes Cortes et al. (2021) find that young, unmarried workers who smoke, have high-risk alcohol consumption, negative self-rated health, or high perceived stress levels exhibit higher levels of ultra-processed food consumption. Finally, Coletro et al. (2022) show that individuals engaging in multiple health-risk behaviors, such as ultra-processed food consumption, sedentary lifestyles, and smoking, are more likely to experience depression and anxiety. However, these studies often rely on self-reported measures, which are prone to recall bias, and rarely establish causality. As a result, we still know little about how naturally occurring stress episodes, such as academic exams, shape actual food purchasing behavior.

Although numerous studies have examined the link between stress and unhealthy eating practices, most existing evidence remains correlational. For instance, in the meta-analysis by Hill et al. (2022), the authors review 54 studies employing a mix of self-reports (surveys, daily diaries), perceived stress (self-report questionnaires), and induced stress (laboratory paradigms). In most of these studies, the relationships documented are correlational rather than causal. Our study contributes to this literature in three main ways. First, it provides causal evidence on the relationship between arguably stressful periods and UPF intake using objective transaction data rather than self-reported measures. Second, it quantifies the behavioral response to exam periods in terms of changes in ultra-processed food purchases for a large university population, allowing us to use non-students as a natural control group. We exploit the fact that the supermarket data include voluntary buyer identification through institutional IDs, which enables us to distinguish students from non-students. Third, by examining a common stressor experienced by millions of students worldwide, this study illustrates how systematic and predictable stressful events can consistently influence dietary behavior, with important implications for nutrition policies and health interventions within academic institutions.

The aim of this paper is to assess whether university students purchase more ultra-processed foods (UPFs) during exam periods compared with non-exam periods. We exploit the unique setting of the Pontificia Universidad Javeriana Cali - Colombia, which hosts a supermarket that simultaneously functions as a research laboratory. We use point-of-sale data from this supermarket to track food purchases made by students and non-students (primarily university employees) between 2018 and 2023. The dataset records every transaction, including product descriptions, quantities, and prices, allowing us to rely on objective purchase information rather than self-reported data to measure UPF consumption. Colombia provides us with an emerging economy context characterized by rapid UPF penetration, but scarce behavioral data. We compare food purchases during exam weeks with those in non-exam weeks for both groups, exploiting the quasi-experimental timing of exams to implement a difference-in-differences framework that estimates the causal effect of exam periods on food choices.

## 2 Materials and methods

### 2.1 Data

To test our hypotheses, we use comprehensive point-of-sale transaction data from the Javeriana Cali Supermarket Lab covering the years 2018, 2019, 2022, and up to August 2023.^1^ This dataset includes detailed information on sales at the primary grocery retailer operating on the university campus^2^, including product ID and description, purchase amount, and the date and time of each transaction. In addition, we are able to identify the customer type for 45.92% of the transactions, with students and university staff constituting the majority of transactions. Students accounted for 74.7% and 54.3% of all purchases in 2018 and 2019, respectively. While the majority of customer identities were unavailable in 2022 and 2023, the pattern of students comprising the largest customer segment persisted, accounting for between 21.6% and 17% of all identified customers. Table 1 shows that the average purchase made by students is lower than the mean purchase made by collaborators.

**Table 1:** Summary statistics of sales by customer type in Colombian pesos.

**Customer type**	**Sum**	**Mean**	**SD**	**Min**	**Max**	**N**
Collaborators	69,786,295	2,302.04	3,674.00	80	136,800	30,315
Students	513,216,230	1,850.71	1,525.71	50	172,800	277,308
Unidentified	743,062,825	2,148.72	2,935.08	80	289,800	345,816

Across all groups, 88.4% of purchased products are classified as ultra-processed foods (UPF) according to the Nova classification. An additional 3.3% of the products did not match the classification database. As reported in Table 2, the average sales value of UPF products is lower than that of minimally processed foods and processed culinary ingredients. This aligns with the economic rationale that UPFs are typically produced using low-cost ingredients to maximize profit margins.

**Table 2:** Summary of sales in Colombian pesos by Nova food classification group.

**Type of product**	**Sum**	**Mean**	**SD**	**Min**	**Max**	**N**
Unprocessed or minimally processed foods	92,485,835	2,276.85	2,567.40	80	150,000	40,620
Oils, fats, salt and sugar	281,900	2,494.69	3,647.28	800	19,900	113
Processed foods	29,156,260	2,026.29	4,400.20	240	195,000	14,389
Ultra-processed foods	1,200,000,000	2,012.55	2,418.39	50	289,800	598,317

### 2.2 Empirical approach

We adopt a difference-in-differences (DiD) framework to estimate the causal effect of exam periods on the consumption of ultra-processed foods. The identification strategy is straightforward: students face exam periods, whereas staff do not. Both groups shop at the same on-campus supermarket and are exposed to the same prices and campus conditions. If exams increase stress and time pressure among students, their purchasing behavior should change during those weeks. Staff serve as a natural comparison group, allowing us to isolate the effect of exams from other concurrent temporal changes.

We compare the same groups, students and staff, during regular weeks and exam weeks, providing a before-and-after comparison for each. The difference between these two changes (the difference of the difference) captures the causal effect of exams. A key assumption behind our approach is that, in the absence of exams, both groups would have followed similar trends in their purchasing behavior over time. This is known as the parallel trends assumption. This assumption allows us to attribute any difference between groups during the exam period to exam-related exogenous shocks rather than to other confounding factors (Angrist and Pischke, 2008). The Difference-in-differences (DiD) helps address the main challenge of causal inference: the fact that we cannot observe what would have happened to the same individuals in the absence of treatment. This method assumes that data are available for a comparison group whose outcomes would have followed the same trend as those of the treated group if the treatment had not occurred, and who are not themselves affected by the treatment.

To estimate the effect of exam periods, we compare how the purchasing behavior of each group changes over time. The first step is to compute the before-and-after difference for students and, separately, the same difference for staff. The second step is to take the difference between these two changes. This two-stage comparison forms the basis of the difference-in-differences method. The change observed among students reflects both the effect of exams and any time-varying factors common to all individuals. In contrast, the change observed among staff captures only the common time component, since staff are not affected by exams. Subtracting the staff change from the student change removes the common component and isolates the causal effect of exams on student purchases.

For the main analysis, we aggregate sales data at the product-week level and classify each product as either ultra-processed or not. We then estimate how exam weeks affect weekly sales using ordinary least squares (OLS) regressions. The specification includes product fixed effects to control for time-invariant product characteristics and week fixed effects to absorb shocks common to all products in a given week. In particular, this analysis implies estimating the following equation:


$$\begin{array}{l} y_{it}=\gamma_{i}+\delta_{t}+\beta_{1}UPF_{i}+\beta_{2}ExamWeek_{t}+\beta_{3}\left(UPF_{i}\times ExamWeek_{t}\right)+\varepsilon_{it}, \end{array}$$ (1)

where the outcome variable *y*_*it*_ denotes the weekly sales (in monetary units) of product *i* during week *t*. The indicator variable *UPF*_*i*_ equals 1 if product *i* is classified as ultra-processed, and 0 otherwise. *ExamWeek*_*t*_ equals 1 if week *t* corresponds to an exam week, and 0 otherwise. The interaction term *UPF*_*i*_×*ExamWeek*_*t*_ (denoted *D*_*it*_) equals 1 for UPF products during exam weeks, defining the treatment condition. Product fixed effects γ_*i*_ control for time-invariant product characteristics, while week fixed effects δ_*t*_ capture time-specific shocks common to all products.

The interaction between the exam week indicator and the ultra processed food indicator gives the effect of exams on ultra processed food sales. The coefficient β_3_ thus represents the difference-in-differences estimate of the impact of exam weeks on UPF sales, which we estimate using ordinary least squares (OLS). Standard errors are clustered at the product level to account for serial correlation in sales over time.

We also estimate models at the customer level, focusing on transactions where the buyer can be identified as either a student or a staff member. In this specification, we compare weekly purchases of ultra-processed foods between students and staff using ordinary least squares (OLS). The equation that represents this analysis is:


$$\begin{array}{l} y_{ict}=\gamma_{i}+\delta_{t}+\beta_{1}Student_{c}+\beta_{2}Exam\text{\_}week_{t}+\beta_{3}D_{ct}+\varepsilon_{ict} \end{array}$$ (2)

where the outcome variable *y*_*ict*_ denotes the weekly sales (in monetary units) of UPF product *i* made by customer group *c* during week *t*. The indicator variable *Student*_*c*_ equals 1 if customer group *c* corresponds to students, and 0 otherwise. *ExamWeek*_*t*_ equals 1 if week *t* is an exam week, and 0 otherwise. The interaction between being a student and the exam week indicator (*D*_*ct*_) captures how students change their purchases during exam weeks relative to staff.^3^

### 2.3 Hypotheses

Ultra-processed foods (UPFs) are defined according to the Nova classification system, which categorizes foods based on the nature, extent, and purpose of the industrial processing they undergo. Nova's definition of food processing encompasses a wide range of physical, biological, and chemical processes applied to foods after they have been separated from their natural state but before they are consumed or used as ingredients in dishes and meals (Monteiro et al., 2018). We use this classification to construct a binary variable distinguishing UPF and non-UPF products.^4^

As mentioned in the introduction, previous research has established a correlation between stress and food consumption, primarily using self-reported measures (Hill et al., 2022). According to Adam and Epel (2007), stress can influence eating behavior in two ways. First, while some individuals tend to eat less and lose weight during or after stressful experiences, most people tend to increase their food intake. Second, stress can trigger cravings for high-calorie foods, particularly among individuals with heightened cortisol responses, thereby contributing to the growing obesity epidemic. The underlying mechanism is associated with the brain's reward system, a network of neural structures involved in reward-related cognition, including pleasure, “wanting,” and motivation. Ulrich-Lai et al. (2015) explain that sweet, palatable foods can alleviate negative emotions by reducing physiological and psychological signs of stress and anxiety following exposure to acute or chronic stressors.

Given that exams are an important source of stress among students, these insights lead us to formulate our main hypotheses. In particular, we expect that the negative shock associated with exam periods increases the consumption of UPF products, leading to Hypothesis 1.

HYPOTHESIS *1. Exposure to exam periods increases students' consumption of ultra-processed foods (UPFs) relative to non-exam periods*.

Most existing studies rely on students' self-reported information or correlate students' eating behavior during stressful periods with that observed during non-stressful periods. However, if Hypothesis 1 holds, we should observe that individuals who are not subject to exam-related effects, or who face the same external conditions unrelated to exams, do not increase their UPF consumption during exam weeks. In other words, exam periods are effectively random dates for non-students and should be orthogonal to their dietary preferences. This reasoning leads to Hypothesis 2.

HYPOTHESIS *2. The effect of exam periods on UPF consumption is larger for students than for university staff or other non-student customers*.

Additionally, since exams represent the external shock that increases UPF consumption, we may also expect the week preceding exams to be similarly stressful and to trigger anxiety as students prepare. If this intuition holds, we should observe anticipatory or preparation-related shocks leading to increased UPF consumption. This rationale is formalized in Hypothesis 3.

HYPOTHESIS *3. Students' consumption of UPF products increases in the week preceding exams, potentially reflecting stress or time constraints associated with exam preparation*.

Finally, once the exam weeks have concluded, and given that these periods are common and mandatory across all academic programs, we can expect that, for instance, students' cortisol levels decrease in the week following exams, leading their dietary habits to return to normal. In other words, if the assumed shock is removed, the associated emotional and behavioral responses should also dissipate. As a consequence, we expect a decline in UPF consumption, as stated in Hypothesis 4.

HYPOTHESIS *4. Students' consumption of UPF products decreases in the week following exams, consistent with the notion that, once negative shocks disappear, the affected population returns to baseline consumption patterns*.

## 3 Results

**Figure 1:**
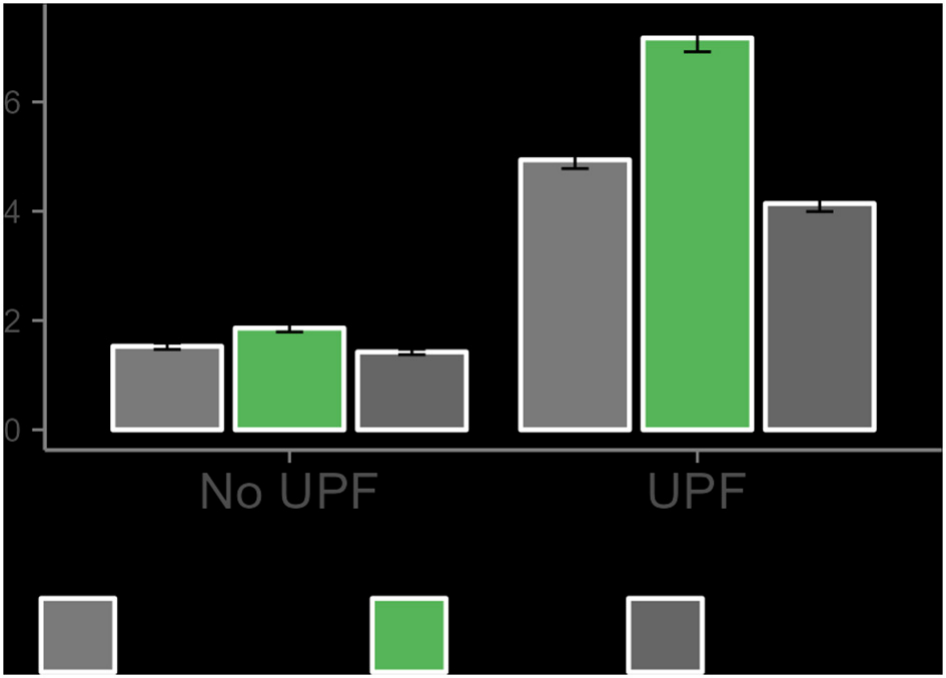
Average purchases by students during exam periods, by food type.

We begin by graphically illustrating the impact of exam weeks on UPF consumption. Figure 1 presents the average number of purchases made by students in the week preceding exams, during exam weeks, and in the week following exams, disaggregated by product type, classified as UPF or non-UPF. Non-UPF products comprise unprocessed or minimally processed foods, oils, processed culinary ingredients, and processed foods. Each bar represents the mean value with its corresponding 95% confidence interval. The figure reveals that the average number of UPF purchases increases during exam weeks relative to the weeks before and after, and this difference is statistically significant at the 95% confidence level. This result is in the expected direction but is informative rather than causal.

### 3.1 Product level analysis

As outlined in the empirical approach section, the first part of the analysis aggregates sales data at the product-week level. We construct weekly aggregates under the assumption that individual purchasing patterns are relatively stable across weeks, though purchases may not occur daily. We classify products into two groups: ultra-processed foods (UPF) and non-ultra-processed foods (non-UPF). We then estimate the impact of exam weeks on weekly UPF sales, using non-UPF products as a control group. We estimate the model represented in Equation 1 using OLS.

Table 3 presents the results of the estimated model described in Equation 1, both with and without product and week fixed effects. Column (2) of Table 3 shows no statistically significant effect of exam weeks for the overall sample of customers (including students, university staff, and unidentified customers). However, when restricting the sample to customers identified as students, we find a statistically significant positive effect of exam weeks on UPF sales of 2,406 COP. This effect corresponds to approximately 12.9% of the mean value of student purchases of non-UPF products during non-exam periods.

**Table 3:** Difference-in-Differences estimates of weekly sales.

	**All sample**		**Student sample**	
	**(1)**	**(2)**	**(3)**	**(4)**
UPF × Exam week	2.686	2.419	2.319**	2.046**
	(2.160)	(2.086)	(0.994)	(1.038)
Exam week	–3.115	9.454***	–3.507***	-8.679***
	(2.148)	(2.467)	(0.982)	(2.054)
UPF	–5.684	6.099**	–4.289	17.213***
	(7.504)	(3.091)	(4.195)	(1.622)
Observations	47,101	47,101	33,106	33,106
Control mean	28.394	28.394	15.924	15.924
Effect size	0.095	0.085	0.146	0.129
Fixed effects	No	Yes	No	Yes

This table reports difference-in-differences estimates of weekly sales (in thousands of COP).

Standard errors are reported in parentheses and are clustered at the product level.

Columns (1)–(2) use the full sample, while Columns (3)–(4) restrict the estimation to student transactions.

Effect sizes are standardized relative to the control mean.

^*^p < 0.10, ^**^p < 0.05, ^***^p < 0.01.

We also examine the effect on the number of UPF product sales. The results, reported in Supplementary Table S1, show a statistically significant positive effect of exam weeks on the number of UPF sales of 1.033 transactions. This effect represents approximately 12.1% of the mean number of student purchases of non-UPF products during non-exam periods, consistent with the results obtained for sales value.

To further explore the effect dynamics, we also estimate the model of Equation 1 taking as treatment indicator the previous and posterior week to exam weeks. Supplementary Tables S2, S3 show the results of these estimations, both for monetary sales and number of sales. We found a statistically significant effect of pre-exam week on UPF sales for all the available sample, but when considering only the student sample, there is no statistically significant effect for both outcome variables. The estimated coefficient is negative but smaller than the one for exam week, representing 1% percent of the students' mean purchases during non-exam periods. This result contributes to the parallel trends assumption, which enables us to argue that exam and non-exam week sales are comparable. However, the fulfillment of the parallel trends cannot be tested because the pre-treatment, in this case, the pre-exam, weeks overlap with the next period of exams.

To further explore the dynamics of the effect, we also estimate the model in Equation 1, redefining the treatment indicator to capture the weeks preceding and following the exam weeks. Supplementary Tables S2, S3 present the results of these estimations for both monetary sales and the number of sales. We find a statistically significant effect of the pre-exam week on UPF sales for the full sample; however, when restricting the sample to students only, the effect is not statistically significant for either outcome variable. The estimated coefficient for the pre-exam week is negative but smaller in magnitude than that for the exam week, representing approximately 1% of the mean student purchases during non-exam periods. This result supports the validity of the parallel trends assumption, suggesting that sales patterns in exam and non-exam weeks are comparable. However, a formal test of the parallel trends assumption is not feasible in this context because the pre-exam weeks partially overlap with the subsequent exam periods. Supplementary Tables S4, S5 present the results for the effect of the post-exam week on monetary sales and on the number of sales. We find no statistically significant effect of the post-exam week indicator on either outcome variable.

### 3.2 Type of customer level analysis

As an alternative specification, we test our hypotheses using the subset of transactions for which the customer type is identifiable and restrict the analysis to products classified as ultra-processed. This specification focuses on the consumption of UPF products; however, it excludes potential substitution effects between UPF and non-UPF products that were captured in the previous analysis, where we found that students tend to purchase more UPF foods during exam weeks compared with non-UPF products. In this specification, we estimate the impact of exam weeks on UPF sales by comparing students (treated group) with non-students (control group). This specification allows us to directly assess whether students purchase more ultra-processed foods than staff during exam weeks, focusing exclusively on UPF transactions from identifiable customers. Product and week fixed effects are included in some specifications to control for time-invariant product characteristics and common time shocks.

Table 4, columns (1, 2), present the estimation results for the effects of exam weeks based on the model described in Equation 2. We find no statistically significant treatment effect for the interaction between the student indicator and the exam week indicator once fixed effects are included. However, the estimated coefficient is positive and represents approximately 2.5% of the mean sales for non-student customers during non-exam periods. Columns (3, 4) of Table 4 report the results for the same specification using the number of sales as the outcome variable. In this case, we find a positive and statistically significant effect of the interaction term, corresponding to an increase of 0.388 sales at the 1% significance level. This effect represents approximately 6.6% of the mean number of sales for non-student customers during non-exam weeks. These results indicate that students make significantly more purchases during exam weeks, even though their total expenditure does not show a statistically significant change, after controlling for product and week fixed effects.

**Table 4:** Exam week effects by client type: Difference-in-Differences estimates for sales value and number of sales.

	**Sales value (thousands COP)**		**Number of sales**	
	**(1)**	**(2)**	**(3)**	**(4)**
Student × Exam week	–0.594***	0.283	–0.292***	0.388***
	(0.172)	(0.240)	(0.080)	(0.135)
Exam week	–0.588***	–4.035***	–0.201***	–6.158***
	(0.099)	(1.321)	(0.023)	(0.490)
Student	11.140***	13.719***	6.464***	8.327***
	(0.532)	(0.757)	(0.275)	(0.395)
Observations	44,614	44,614	44,614	44,614
Control mean	11.190	11.190	5.894	5.894
Effect size	–0.053	0.025	–0.050	0.066
Fixed effects	No	Yes	No	Yes

This table reports difference-in-differences estimates of student behavior during exam weeks.

Columns (1)–(2) use the total sales value (in thousands of COP) as the dependent variable, while Columns (3)–(4) use the number of sales.

Standard errors are reported in parentheses and clustered at the product level.

Effect sizes are standardized relative to the control mean.

^*^p < 0.10, ^**^p < 0.05, ^***^p < 0.01.

Table 5 presents the results using the post-exam week as the treatment indicator. The results show a negative and statistically significant effect of the interaction term (Student × Post-exam week) on both monetary sales and the number of sales. This finding indicates that UPF sales among students decrease in the week following exams, representing 25.7% of the mean UPF sales value and 27.7% of the mean number of UPF products sold during non-post-exam weeks. These results are consistent with the hypothesis that, once the exam period concludes, students reduce their consumption of UPF products.

**Table 5:** Post exam week effects by client type: difference-in-differences estimates for sales value and number of sales.

	**Sales value (thousands COP)**		**Number of sales**	
	**(1)**	**(2)**	**(3)**	**(4)**
Student × Post-exam week	–3.092***	–3.033***	–1.522***	–1.736***
	(0.250)	(0.346)	(0.128)	(0.195)
Post-exam week	–0.014	-5.630***	–0.094***	–4.722***
	(0.145)	(0.908)	(0.033)	(0.496)
Student	11.317***	14.162***	6.555***	8.644***
	(0.536)	(0.772)	(0.283)	(0.412)
Observations	44,614	44,614	44,614	44,614
Control mean	11.807	11.807	6.274	6.274
Effect size	–0.262	–0.257	–0.243	–0.277
Fixed effects	No	Yes	No	Yes

This table reports difference-in-differences estimates of post-exam week effects on student sales behavior at the client level.

Columns (1)–(2) use the total sales value (in thousands of COP) as the dependent variable, while Columns (3)–(4) use the number of sales.

Standard errors are in parentheses and clustered at the product level.

Effect sizes are standardized relative to the control mean.

^*^p < 0.10, ^**^p < 0.05, ^***^p < 0.01.

### 3.3 Type-of-client analysis treating unidentified clients as students

**Figure 2:**
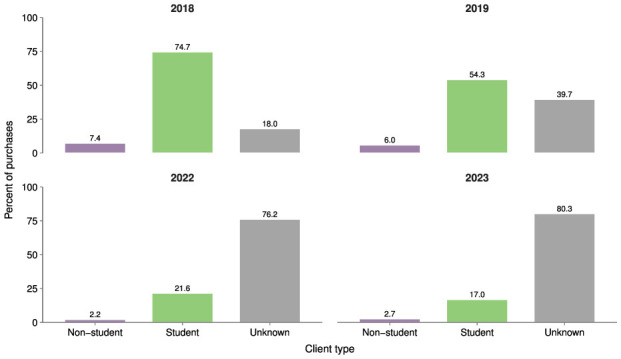
Share of sales by customer type and year.

In the Javeriana Cali Supermarket Lab, when a customer makes a purchase, they can choose whether to present their institutional ID, provide their ID number, or not provide identification at all. This procedure allows us to accurately identify whether a buyer is a student when the ID is presented. However, over time, this identification process became less reliable. Some customers may have chosen not to provide their IDs, or staff members may have found the procedure time-consuming and therefore recorded purchases without an ID. Figure 2 presents the distribution of identifiable customer types, showing that in 2022 and 2023 the proportion of student transactions declined substantially, while the share of unidentified customers increased. Because a considerable share of total sales are made by unidentified clients, this could potentially affect the results presented above.

To address this issue, we conduct a robustness check by re-estimating the client-level models presented in the previous section, assuming that all unidentified clients are students. Given that the majority of the university population consists of students, this assumption is arguably conservative and likely leads to an underestimation of the true effects, relative to assuming that only a fraction of the unidentified clients are students. Table 6 presents a positive and statistically significant effect of the interaction between the student indicator and the exam week indicator: an increase of 1,769 COP in monetary sales and 1.396 in the number of sales. The effect remains statistically significant and corroborates the results presented in the previous section.

**Table 6:** Exam-week effects on sales and number of sales at the client level, assuming unidentified clients are students.

	**Sales value (thousands COP)**		**Number of sales**	
	**(1)**	**(2)**	**(3)**	**(4)**
Student × Exam week	0.320	1.769***	0.456***	1.396***
	(0.237)	(0.368)	(0.114)	(0.205)
Exam week	–0.588***	7.268***	–0.201***	–2.067***
	(0.099)	(1.599)	(0.023)	(0.622)
Student	22.304***	25.797***	11.292***	14.665***
	(1.073)	(1.485)	(0.506)	(0.703)
Observations	56,682	56,682	56,682	56,682
Control mean	19.779	19.779	9.739	9.739
Effect size	0.016	0.089	0.047	0.143
Fixed effects	No	Yes	No	Yes

*Note:* This table presents difference-in-differences estimates of exam week effects by client type at the individual level, assuming unidentified clients are students.

Columns (1)–(2) use total sales value (in thousands of COP) as the dependent variable, while Columns (3)–(4) use the number of sales.

The interaction term between the student indicator and exam week captures differences in sales behavior relative to non-students during regular weeks.

Standard errors are reported in parentheses and clustered at the product level. ^*^p < 0.10, ^**^p < 0.05, ^***^p < 0.01.

On the other hand, Table 7 presents a positive and statistically significant effect of the interaction between the student indicator and the pre-exam week indicator: an increase of 2,774 COP in monetary sales and 0.002 in the number of sales. This effect is strong and statistically significant, potentially indicating that, once the larger sample is considered, students already experience elevated stress or constraints in the week preceding exams, possibly due to preparation activities. If this interpretation is correct, UPF consumption should decrease in the weeks following exams. This is precisely what Table 8 shows in the interaction term: a negative and statistically significant effect of the interaction between the student indicator and the post-exam week indicator, corresponding to a decrease of 3,799 COP in monetary sales and 2.016 in the number of sales. These results suggest that once the exam period concludes, students substantially reduce their consumption of UPF products.

**Table 7:** Pre-exam week effects on sales and number of sales at the client level, assuming unidentified clients are students.

	**Sales value (thousands COP)**		**Number of sales**	
	**(1)**	**(2)**	**(3)**	**(4)**
Student × Pre-exam week	3.017***	2.774***	0.001***	0.002***
	(0.330)	(0.480)	(0.000)	(0.000)
Pre-exam week	–0.512***	–9.125***	–0.000**	–0.000
	(0.111)	(1.548)	(0.000)	(0.001)
Student	21.978***	25.925***	0.011***	0.015***
	(1.054)	(1.489)	(0.001)	(0.001)
Observations	56,682	56,682	56,682	56,682
Control mean	20.363	20.363	0.010	0.010
Effect size	0.148	0.136	0.113	0.151
Fixed effects	No	Yes	No	Yes

*Note:* This table presents difference-in-differences estimates of pre-exam week effects by client type at the individual level, assuming unidentified clients are students.

Columns (1)–(2) use total sales value (in thousands of COP) as the dependent variable, while Columns (3)–(4) use the number of sales.

The interaction term between the student indicator and pre-exam week captures differences in purchasing behavior relative to non-students during regular weeks.

Standard errors are reported in parentheses and clustered at the product level.

^*^p < 0.10, ^**^p < 0.05, ^***^p < 0.01.

**Table 8:** Post-exam week effects on sales and number of sales at the client level, assuming unidentified clients are students.

	**Sales value (thousands COP)**		**Number of sales**	
	**(1)**	**(2)**	**(3)**	**(4)**
Student × Post-exam week	–2.389***	–3.799***	–1.118***	–2.016***
	(0.336)	(0.707)	(0.166)	(0.353)
Post-exam week	–0.014	0.404	–0.094***	–2.620***
	(0.145)	(1.441)	(0.033)	(0.679)
Student	22.673***	26.733***	11.554***	15.288***
	(1.092)	(1.536)	(0.520)	(0.740)
Observations	56,682	56,682	56,682	56,682
Control mean	20.911	20.911	10.393	10.393
Effect size	–0.114	–0.182	–0.108	–0.194
Fixed effects	No	Yes	No	Yes

*Note:* This table presents difference-in-differences estimates of post-exam week effects by client type at the individual level, assuming unidentified clients are students.

Columns (1)–(2) use total sales value (in thousands of COP) as the dependent variable, while Columns (3)–(4) use the number of sales.

The interaction term between the student indicator and post-exam week captures differences in purchasing behavior relative to non-students during regular weeks.

Results show a negative and statistically significant effect on both monetary sales and the number of sales, indicating a reduction in purchases after exam weeks.

Standard errors are reported in parentheses and clustered at the product level.

^*^p < 0.10, ^**^p < 0.05, ^***^p < 0.01.

## 4 Discussion

Improper eating behaviors among university students pose long-term risks to both physical and mental health. High consumption of processed and nutrient-poor foods is associated with increased likelihood of obesity and chronic conditions. Studies report higher BMI, greater waist circumference, and elevated risks of cardiovascular disease and type 2 diabetes among students who frequently consume fast foods and sweets (Neri et al., 2022; Du et al., 2024; Abdurasulova, 2025). Poor dietary choices can also result in nutritional deficiencies that impair immune function and heighten susceptibility to infectious diseases (Peng et al., 2023). These patterns extend to mental health: disordered eating behaviors often emerge as stress-coping mechanisms and are linked to elevated psychological distress (Kärkkäinen et al., 2018; José et al., 2025). Nutrient deficiencies and unhealthy diets have also been shown to reduce attention, memory, and overall cognitive function, with adverse effects on academic performance (Jackson et al., 2015). Stress, time constraints, financial limitations, and the campus food environment all shape student dietary behavior. Limited access to healthy options can further entrench poor eating habits.

This study provides quasi-experimental evidence that exam periods significantly alter food purchasing behavior among university students. Using objective transaction data from a campus supermarket at a large Colombian university, we find that students increase their purchases of ultra-processed foods (UPFs) by 12.9% during exam weeks relative to non-exam periods. Importantly, this effect is not observed among university staff, who shop at the same location during the same periods. This strengthens confidence that the observed changes among students reflect exam-specific influences rather than broader seasonal or environmental factors affecting the entire campus community. This result is consistent with Hypothesis 1, 2 and reinforces the conclusion that predictable academic stressors have a measurable impact on students' purchasing behavior. More importantly, this finding extends previous correlational evidence (Karunanayake et al., 2020; Michels et al., 2020; Mohamed et al., 2020; Faixová et al., 2023) by establishing a causal relationship using objective transaction data.

The contrasting responses observed during examination periods between students and university staff members (who do not take exams) reinforce the causal validity of our findings by showing that the observed effects are limited to individuals undergoing exam-induced negative shocks. This naturally occurring comparison group approach successfully eliminates competing explanations, including seasonal fluctuations, modifications in campus food options, or other time-related variables that might influence the entire university population uniformly. Understanding why students increase UPF purchases during exam periods requires consideration of multiple, potentially overlapping behavioral mechanisms. First, according to the psychobiological model of stress and eating (Adam and Epel, 2007; Gibson, 2012), acute stressors activate the hypothalamic–pituitary–adrenal (HPA) axis, leading to elevated cortisol production. Higher cortisol levels are associated with increased cravings for calorie-dense, highly palatable foods–characteristics typical of many ultra-processed products. This physiological pathway suggests that students may engage in “comfort eating,” using UPFs as a hedonic coping strategy to temporarily alleviate the negative emotional states induced by exam-related anxiety.

Beyond the main effect, our analysis uncovers a distinct temporal pattern in purchasing behavior. Students increase UPF purchases by 2,774 COP in the week preceding exams and reduce purchases by 3,799 COP in the week following exams. This full cycle: anticipatory increase, exam-week elevation, and post-exam normalization, constitutes one of the study's most novel contributions. While prior research has documented stress-related dietary changes during acute stressors (Hill et al., 2022), few studies have captured the complete temporal trajectory surrounding predictable stress events using objective behavioral data. The brain's reward system offers a mechanistic explanation for this behavior. Ultra-processed foods, engineered to be hyper-palatable through specific combinations of sugar, fat, and salt, stimulate dopaminergic reward pathways more intensely than minimally processed alternatives (Monteiro et al., 2018). Under stress, individuals may seek stronger reward stimuli as a compensatory response to negative affect, increasing the appeal of ultra-processed products. Our finding that UPF purchases increase before exams and decrease after exams aligns with this emotional regulation framework. Additionally, this pattern aligns with the allostatic load model, which posits that anticipatory stress responses can be as physiologically and behaviorally consequential as the stressor itself (McEwen, 2007). The pre-exam increase likely reflects both anticipatory anxiety and time constraints, which make convenient, highly palatable foods more attractive than healthier alternatives.

One implication of these findings is that, from a cognitive performance perspective, the consumption of UPFs during stressful academic weeks may be counterproductive. While such foods offer immediate benefits like comfort, stress reduction, and ease of preparation, they can simultaneously trigger unstable blood glucose levels, diminished mental capacity, and emotional volatility, all of which may undermine academic achievement and intensify stress responses (Gómez-Pinilla, 2008). Consequently, educational institutions should prioritize developing programs that encourage effective stress management techniques and nutritious dietary practices throughout high-pressure academic phases. Significantly, these programs must extend beyond examination periods to include the preparatory weeks leading up to them, considering the anticipatory stress responses identified in this research. This temporal pattern offers a clear window of opportunity for preventive interventions aimed at improving both student well-being and academic outcomes.

This study addresses several key limitations in the existing literature. In particular, the use of objective point-of-sale transaction data with customer identification type eliminates recall bias and social desirability effects common in self-report studies. The quasi-experimental design, arising from externally imposed exam schedules, enables causal inference without the ethical concerns associated with experimentally inducing stress among students or the artificiality of doing so. Employing a difference-in-differences framework with non-student controls constitutes a notable methodological contribution to this literature. By comparing students (treatment group) and university staff (control group) across exam and non-exam periods, we isolate the effect of exam-related negative shocks from other temporal shocks affecting the university community.

Several important limitations should be considered when interpreting our findings. First, the data capture purchasing behavior, not actual consumption. Students may purchase UPFs without consuming them, share items with peers, or stockpile products for future use. This distinction is critical: while purchasing patterns reflect behavioral intentions and resource allocation decisions, they remain imperfect proxies for true dietary intake. This limitation is partially mitigated by the setting: a campus supermarket where students tend to make frequent, relatively small purchases (mean purchase value of 1,850 COP), rather than engaging in bulk, weekly shopping. This pattern suggests that purchases are likely intended for immediate or near-term consumption, rather than stockpiling. Nevertheless, in the absence of dietary recall data or biomarkers of UPF intake, we cannot directly confirm that purchased items were consumed. Second, although our difference-in-differences approach provides credible causal inference, we cannot directly test the parallel trends assumption. Ideally, we would demonstrate that students and staff exhibited parallel purchasing trends prior to exam periods, supporting the assumption that both groups would have followed similar trajectories in the absence of treatment. However, testing this assumption is complicated by the fact that pre-exam weeks partially overlap with the end of previous exam cycles, making it difficult to identify a clean pre-treatment baseline. While some specifications show no statistically significant differences in the pre-exam period for students, this does not constitute a formal test of the parallel trends assumption. Future research could extend baseline observation periods to more clearly establish pre-treatment trends.

Third, the findings are based on data from a single university, which limits their external validity. Institutions in different cultural or economic contexts may exhibit different responses to exam periods. However, the underlying phenomenon, that predictable academic stressors influence food purchasing behavior, is likely to be broadly generalizable, even if the magnitude of the effect is context-dependent. Finally, the study does not include physiological measures of stress or comparison groups outside the university setting. While collecting such data was not feasible in this context, incorporating them in future research would allow for a more comprehensive assessment of the relationship between stress and dietary choices. Future research should incorporate physiological measures of stress, such as cortisol levels during exam weeks, to examine their impact with UPF consumption.

The increase in ultra-processed food purchases during exam periods highlights that universities face predictable windows of nutritional vulnerability. Institutions can respond by treating exams, and the weeks leading up to them, as periods requiring targeted support (Almoraie et al., 2024). Effective interventions may include expanding access to stress management services, improving the availability of convenient, exercise, healthy food options, and adjusting academic workloads or schedules that exacerbate cognitive load (Baghurst and Kelley, 2014; Koschel et al., 2017; Reschke et al., 2024). These strategies directly address the emotional strain, mental fatigue, and time constraints that influence students' dietary choices during high-stress periods.

A second implication concerns timing. Our findings show that changes in food purchasing behavior begin prior to the start of exams, suggesting that early interventions may be more effective than support limited to exam week itself. Universities could introduce practical preparatory initiatives in the preceding week, such as meal planning workshops, time management guidance, and the distribution of healthy snack options (Roy et al., 2015; Li et al., 2022). Even if exam-related dietary shifts are temporary, their occurrence during a period when cognitive performance is especially critical underscores the value of targeted, low-cost interventions that support healthier choices during this high-stakes window.

## Data Availability

The datasets presented in this study can be found in online repositories. The names of the repository/repositories and accession number(s) can be found below: https://osf.io/fp3g2.

## Footnotes

1 The analysis relies on administrative records from a single campus supermarket and does not include an ex ante sample size calculation in the conventional survey sense, as the dataset comprises the full universe of recorded transactions by students and staff.

2 Although the on-campus data do not fully capture academic staff's purchasing behavior, or its impact may be different to student population, this limitation does not affect the identification strategy. The difference-in-differences approach relies on temporal changes rather than absolute levels of purchasing. Staff experience the same campus conditions and the same time shocks as students, but they are not exposed to exam periods. Their on-campus purchasing behavior serves as a valid counterfactual for the trend that would have occurred among students absent the exam period. What matters is that exam dates do not influence staff behavior, which is consistent with the data.

3 Exam weeks correspond to official university assessment periods identified through the academic calendars for each semester. Each academic year typically includes two or three exam weeks per semester, corresponding to midterm and final evaluations. For example, in 2022-1, exams took place between February 21 and March 5 (midterm 1), April 4 to April 9 and April 18 to April 23 (midterm 2, divided by Semana Santa), and May 23 to June 4 (finals). The variable “examweek” equals 1 if the date of a transaction falls within any of these periods, and 0 otherwise.

4 A practical way to identify an ultra-processed product is to check whether its list of ingredients includes at least one item characteristic of the Nova ultra-processed food group—namely, food substances never or rarely used in household kitchens (such as high-fructose corn syrup, hydrogenated or interesterified oils, and hydrolyzed proteins) or classes of additives designed to make the final product palatable or more appealing (such as flavors, flavor enhancers, colors, emulsifiers, emulsifying salts, sweeteners, thickeners, and anti-foaming, bulking, carbonating, foaming, gelling, and glazing agents).
