# Supplementary material for: Academic exam periods and ultra-processed food consumption: evidence from supermarket transactions in a Colombian University
Source: Front Psychol. 2026 Jan 27;16:1726856. doi: 10.3389/fpsyg.2025.1726856 (PMC12888042; doi:10.3389/fpsyg.2025.1726856)
Supplement: Supplementary file 1 [file Data_Sheet_1.pdf]

Supplementary Material

1 SUPPLEMENTARY TABLES AND FIGURES

Table S1. Difference-in-Differences estimates of weekly sales by product type.

	All sample		Student sample	
	(1)	(2)	(3)	(4)
UPF × EXAM WEEK	1.416	1.303	1.037***	1.033**
	(0.886)	(0.851)	(0.392)	(0.412)
EXAM WEEK	-1.187	-2.087**	-1.537***	-9.364***
	(0.878)	(0.997)	(0.383)	(0.797)
UPF	-2.304	4.456***	-1.780	12.929***
	(3.141)	(1.361)	(1.830)	(0.910)
Observations	47,101	47,101	33,106	33,106
Control mean	13.876	13.876	8.567	8.567
Effect size	0.102	0.094	0.121	0.121
Fixed effects	No	Yes	No	Yes

Note: This table presents difference-in-differences estimates of weekly sales by product type (in thousands of COP). Standard errors are reported in parentheses and clustered at the product level. Columns (1)–(2) use the full sample, while Columns (3)–(4) restrict the estimation to student transactions. Effect sizes are standardized relative to the control mean. * $p < 0.10$, ** $p < 0.05$, *** $p < 0.01$.

Table S2. Difference-in-Differences estimates of weekly sales before exams.

	All sample		Student sample	
	(1)	(2)	(3)	(4)
UPF × PRE EXAM WEEK	3.531***	2.301**	0.521	-0.156
	(0.817)	(1.006)	(0.747)	(0.886)
PRE EXAM WEEK	-1.241	-13.469***	-0.867	9.780***
	(0.763)	(2.219)	(0.728)	(1.543)
UPF	-5.403	5.396*	-3.674	17.217***
	(7.216)	(3.117)	(4.012)	(1.623)
Observations	47,101	47,101	33,106	33,106
Control mean	27.919	27.919	15.588	15.588
Effect size	0.126	0.082	0.033	-0.010
Fixed effects	No	Yes	No	Yes

Note: This table reports difference-in-differences estimates of weekly sales in the week preceding exams (in thousands of COP). Standard errors are reported in parentheses and are clustered at the product level. Columns (1)–(2) use the full sample, while Columns (3)–(4) restrict the estimation to student transactions. Effect sizes are standardized relative to the control mean. * $p < 0.10$, ** $p < 0.05$, *** $p < 0.01$.

Table S3. Difference-in-Differences estimates of the number of sales before exams.

	All sample		Student sample	
	(1)	(2)	(3)	(4)
UPF × PRE EXAM WEEK	0.971**	0.543	0.256	0.216
	(0.439)	(0.494)	(0.388)	(0.492)
PRE EXAM WEEK	0.075	-0.334	-0.412	8.016***
	(0.415)	(0.983)	(0.376)	(0.867)
UPF	-2.032	4.293***	-1.508	12.930***
	(3.049)	(1.379)	(1.782)	(0.911)
Observations	47,101	47,101	33,106	33,106
Control mean	13.774	13.774	8.427	8.427
Effect size	0.070	0.039	0.030	0.026
Student sample	No	No	Yes	Yes
Fixed effects	No	Yes	No	Yes

Note: This table presents difference-in-differences estimates of the number of sales in the week preceding exams (in thousands of COP). Standard errors are reported in parentheses and clustered at the product level. Columns (1)–(2) use the full sample, while Columns (3)–(4) restrict the estimation to student transactions. Effect sizes are standardized relative to the control mean. * $p < 0.10$, ** $p < 0.05$, *** $p < 0.01$.

Table S4. Difference-in-Differences estimates of weekly sales after exams.

	All sample		Student sample	
	(1)	(2)	(3)	(4)
UPF × POST EXAM WEEK	0.945	0.098	-0.292	-0.671
	(1.470)	(1.257)	(0.767)	(0.862)
POST EXAM WEEK	-3.228**	-3.437	-2.828***	-11.875***
	(1.425)	(2.188)	(0.730)	(1.616)
UPF	-5.043	6.101**	-3.571	17.256***
	(7.356)	(3.076)	(4.054)	(1.617)
Observations	47,101	47,101	33,106	33,106
Control mean	28.448	28.448	15.884	15.884
Effect size	0.033	0.003	-0.018	-0.042
Fixed effects	No	Yes	No	Yes

Note: This table reports difference-in-differences estimates of weekly sales in the week following exams (in thousands of COP). Standard errors are reported in parentheses and clustered at the product level. Columns (1)–(2) use the full sample, while Columns (3)–(4) restrict the estimation to student transactions. Effect sizes are standardized relative to the control mean. * $p < 0.10$, ** $p < 0.05$, *** $p < 0.01$.

Table S5. Difference-in-Differences estimates of the number of sales after exams.

	All sample		Student sample	
	(1)	(2)	(3)	(4)
UPF × POST EXAM WEEK	0.482	0.074	-0.228	-0.603
	(0.558)	(0.441)	(0.397)	(0.406)
POST EXAM WEEK	-1.661***	-5.799***	-1.390***	-8.449***
	(0.532)	(0.948)	(0.373)	(0.847)
UPF	-1.964	4.456***	-1.447	12.966***
	(3.106)	(1.360)	(1.796)	(0.910)
Observations	47,101	47,101	33,106	33,106
Control mean	14.032	14.032	8.585	8.585
Effect size	0.034	0.005	-0.027	-0.070
Fixed effects	No	Yes	No	Yes

Note: This table reports difference-in-differences estimates of the number of sales in the week following exams (in thousands of COP). Standard errors are reported in parentheses and clustered at the product level. Columns (1)–(2) use the full sample, while Columns (3)–(4) restrict the estimation to student transactions. Effect sizes are standardized relative to the control mean. * $p < 0.10$, ** $p < 0.05$, *** $p < 0.01$.
